# Intelligent long-term performance analysis in power electronics systems

**DOI:** 10.1038/s41598-021-87165-3

**Published:** 2021-04-06

**Authors:** Saeed Peyghami, Tomislav Dragicevic, Frede Blaabjerg

**Affiliations:** 1 Department of Energy Technology, Aalborg University, 9220 Aalborg, Denmark; 2 Department of Electrical Engineering, Technical University of Denmark, 2800 Kgs. Lyngby, Denmark

**Keywords:** Energy science and technology, Electrical and electronic engineering, Engineering, Energy infrastructure, Energy grids and networks

## Abstract

This paper proposes a long-term performance indicator for power electronic converters based on their reliability. The converter reliability is represented by the proposed constant lifetime curves, which have been developed using Artificial Neural Network (ANN) under different operating conditions. Unlike the state-of-the-art theoretical reliability modeling approaches, which employ detailed electro-thermal characteristics and lifetime models of converter components, the proposed method provides a nonparametric surrogate model of the converter based on limited non-linear data from theoretical reliability analysis. The proposed approach can quickly predict the converter lifetime under given operating conditions without a further need for extended, time-consuming electro-thermal analysis. Moreover, the proposed lifetime curves can present the long-term performance of converters facilitating optimal system-level design for reliability, reliable operation and maintenance planning in power electronic systems. Numerical case studies evaluate the effectiveness of the proposed reliability modeling approach.

## Introduction

Long-term performance and reliability of a power electronic converter have gained increasing interest for optimal design and operation of power electronic systems^1^. So far, the short-term characteristics of converters, such as fault ride through capability, and voltage/frequency/power quality support, have been taken into account for design and manufacturing, which are also covered by some standards such as IEC 62109^2^ and IEEE 1547^3^. However, weak long-term performance of a converter will introduce more operational and maintenance costs compared to the manufacturing costs^4,5^. This is due to the fact that the power electronic converters contain failure-prone parts such as power devices which are also prone to wear-out failures according to the field experience^6^. Therefore, reliability modeling in modern power electronic based power systems is of paramount importance.

In the conventional power engineering, the reliability is modeled based on historical data provided by the system operator or handbooks such as MIL 217^7^. However, these approaches suffer from poor accuracy. Moreover, they have failed to identify the weakest points of converters, thus not suitable for reliability reinforcement^8^. Due to this fact, physics of failure analysis based approaches have been introduced in recent decade to assess and enhance the reliability of converters^1,9–15^. Meanwhile, these approaches are still the method of choice used in power system reliability engineering. In recent decade, in power electronics engineering model-based reliability analysis on the basis of physics of failures is developed to model the impact of different failure sources and mechanisms on the reliability of converters. According to this method, Stress-Strength Analysis (SSA) is performed to obtain the aging probability of converter components taken into account the various uncertainties. The models used for strength of components are obtained either empirically or theoretically^6,16,17^, which are modeling the aging process of power devices^7^. The validation of the models for converter reliability prediction with power cycling tests has been addressed in the literature e.g., in^16^. In fact, physics of failure analysis is one step ahead of power system reliability engineering, which just relied on historical data without considering the mission-profile based aging of converters.

This concept has been widely applied for photovoltaic and wind power applications^1,6,18^ in order to design the converter or improve its reliability under a given mission profile. In general applications such as battery storage systems, electric vehicle chargers, interlinking converters in micro-grids, and multi-terminal DC grids, such converters are facing different operating conditions depending on power system demand and generation. Thereby, following the grid generation and demand, the converter operating condition will be different. As a result, estimating the converter lifetime for various operating conditions can be time-consuming considering the electro-thermal modeling and Monte Carlo analysis^7,18–22^. Moreover, power networks are complicated systems of systems that include huge number of components. Using stress-strength analysis for reliability modeling for each converter is not possible in practice. This is due to the fact that the SSA requires to have detailed electro-thermal characteristics and lifetime models of the converter components. Furthermore, in the most cases, the detailed electro-thermal models and applied control systems are not provided by manufacturers. Therefore, in order to have optimal design, planning and operation in modern power systems, an accurate, at the same time, simple reliability models for converters are essential.

This is the main motivation to introduce a new modeling approach for reliability of power electronic converters as one of the most failure prone component in power systems, where AI is deployed to simplify the stress-strength analysis and make it computationally feasible when a large number of components is considered. AI is used as a surrogate lifetime estimator where there is no need for detailed electro-thermal and stress-strength analysis for power system-level applications. This will facilitate rapid reliability analysis in power systems with large number of components. This paper presents how to use model-based reliability approaches with the help of AI for power system reliability analysis. It is worth to mention that the reliability of power converters has been deeply addressed in power electronic engineering, while in system-level, i.e., power system engineering, model-based reliability analysis is in the early stage. This is due to the fact that power system is a complicated system and model-based approaches are very time consuming and are not applicable for these systems. Using simplified model will help to incorporate model based analysis in power system engineering. Using model-based approaches helps to have accurate model for reliability assessment and enhancement based on the physics of failures, ultimately leading to optimal decision-makings.

Therefore, this paper proposes a new reliability modeling approach for power electronic converters by introducing a new lifetime-based performance indicator for converters. The converter reliability is represented by constant lifetime curves. These curves cover the entire operating span of a converter in the active-reactive power plane. In order to obtain these lifetime curves, an Artificial Neural Network (ANN) is employed as a nonparametric surrogate model for a converter performance indicator. Hence, it models the converter reliability precisely without using detailed converter component information. In the first step, the converter reliability is estimated in limited operating points using the SSA. Notably, these data can be provided by manufacturers for limited points on the proposed curves as well. Then the ANN is used as a nonparametric surrogate model of the converter to quickly estimate the constant lifetime curves for the entire operating conditions. Furthermore, the proposed curves as the power converter performance index are used for estimating the converter lifetime under different operating mission profiles without using any detailed electro-thermal models and SSA. As a result, the proposed ANN-based approach can facilitate the lifetime prediction of the converters in order to provide a wide range of lifetime curves for different operating conditions. Furthermore, the proposed reliability curves with the help of ANN can be used at system-level for an optimal design for reliability, optimal operation and maintenance planning in PEPSs.

In the following, the proposed power converter long-term performance indicator is explained in “Proposed long-term lifetime performance indicator”. The converter reliability prediction approach based on electro-thermal analysis is explained in “Reliability prediction in converters”. Furthermore, “Proposed AI-based reliability prediction” presents the proposed AI-based reliability modeling method and its application in predicting converter performance indicator. Numerical analysis is provided in “Numerical analysis” illustrating the effectiveness of the proposed method. Moreover, the system level application of proposed AI-based indicator in reliable operation of a PEPS is demonstrated in “Power system-level application”. Finally, the outcomes are summarized in “Power system-level application” including future perspectives.

## Proposed long-term lifetime performance indicator

A power electronic converter is usually designed for a specific application with specific input, output, and general characteristics such as input/output voltage, operating frequency, rated power, reactive power support, total harmonic distortion and efficiency. These characteristics indicate the short-term data provided by manufacturers, while the long-term performance of a converter—e.g., its reliability—may not be provided in the datasheets. So far, there is no standard for long-term performance testing and measuring of converters unlike the short-term performance^23^. Moreover, the converter reliability depends on the operating and environmental conditions. The converter loading, ambient temperature, humidity and vibration are also some of the factors affecting the lifetime of the converter components. Therefore, unlike the short-term indicators, the reliability cannot be presented by just a single number in a converter datasheet.

However, the converter lifetime is a key factor affecting the initial investment and the economic analysis of a project where its replacement and maintenance costs might become higher than the manufacturing costs. In practice, especially during planning, operation and maintenance scheduling, converter reliability behavior is of paramount importance. Therefore, providing lifetime characteristics of converters by manufacturers can facilitate planning, economical decision-making, operational planning, maintenance scheduling and system-level design for reliability in PEPSs.

**Figure 1:**
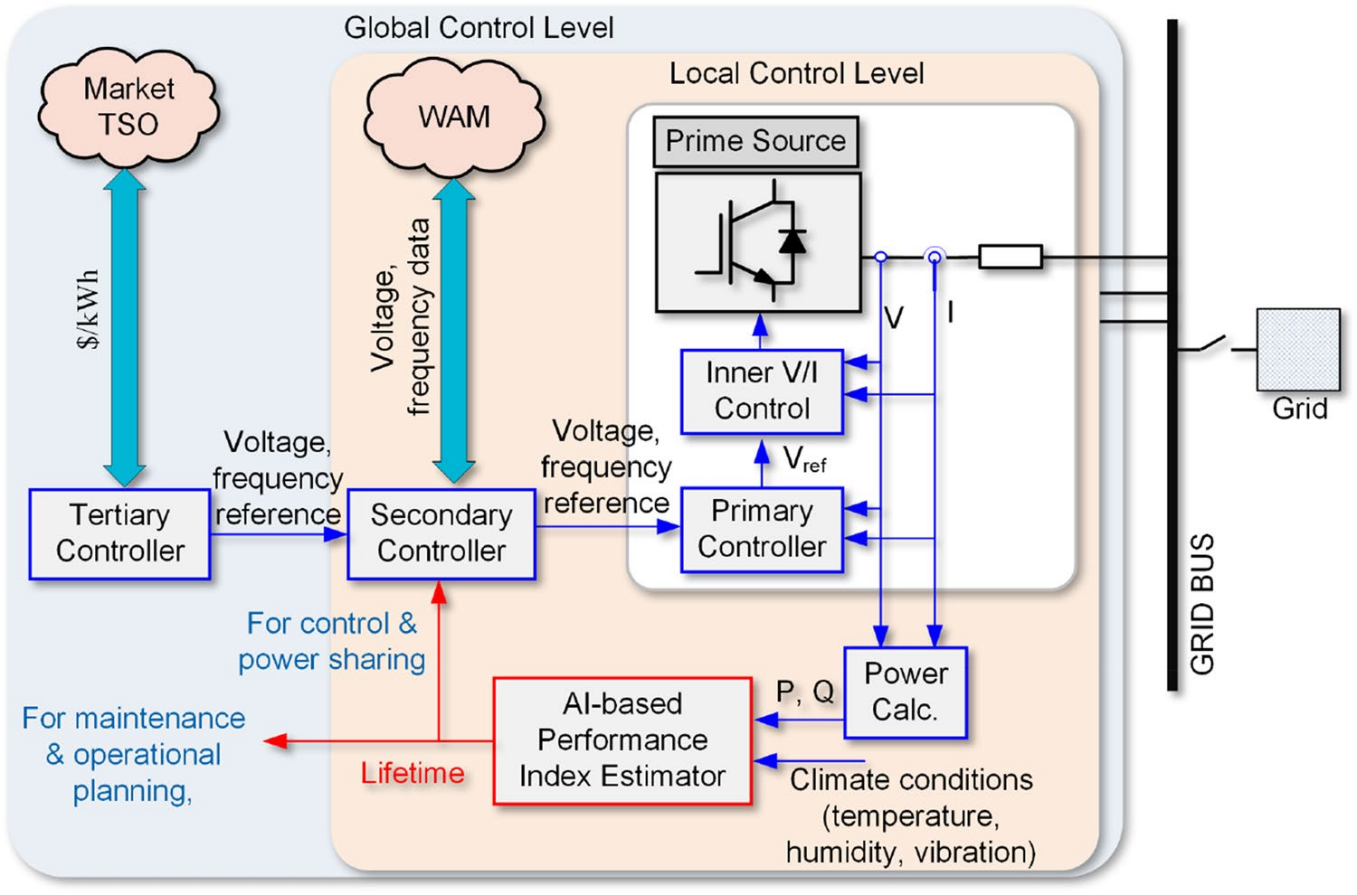
Hierarchical control structure of a power converter with proposed AI-based performance index estimator.

For instance, if the converter performance index is provided, its lifetime can be predicted according to operation and climate conditions estimation. Hence, the obtained lifetime can be used for system-level planning, operation and design for reliability purposes. Moreover, this indicator can be implemented in the converter control system as shown in Fig. 1. Using the operating condition and climate data within a specified time period, e.g., every month, the performance indicator can be estimated. Hence, this indicator can be used to monitor the converter lifetime and managing its lifetime. This can be performed by power-sharing control^24^ for adjusting the converter loading. Moreover, the performance indicator can be used within operational planning and maintenance scheduling. The converter lifetime can be used for preventive maintenance activities, which can facilitate the optimal operation of converters in power systems. In practice, a converter can be used for different applications. Even if it is used for an identical application, the operating conditions are not necessarily identical. For instance, solar inverters experience different loading due to climate conditions (e.g., irradiance and temperature). Moreover, loading of an HVDC converter between two areas varies, or an interlinking converter in microgrids depending on demands and energy management strategies. The same arguments are valid for other applications such as motor drives, electric vehicle chargers, and so on.

As already mentioned, there are different factors affecting the converter lifetime. A converter failure can occur due to random or systematic causes^19^. Systematic failures have non-physical causes such as design error, installation error, etc. Moreover, the random failures happen in a random time due to one or more degradation mechanisms in hardware. Random hardware failure may be induced by overstressing or aging of components^19^. The first one is known as random chance failures which depend on a sudden change in operating conditions such as grid faults. This could be modeled by historical field-return reliability data. However, the random wear-out failures depend on the physical strength of components and applied stress to the potential failure cites of components. Therefore, wear-out failure can be modeled by stress-strength analysis for all the converter fragile components^17,19,20,25^.

**Figure 2:**
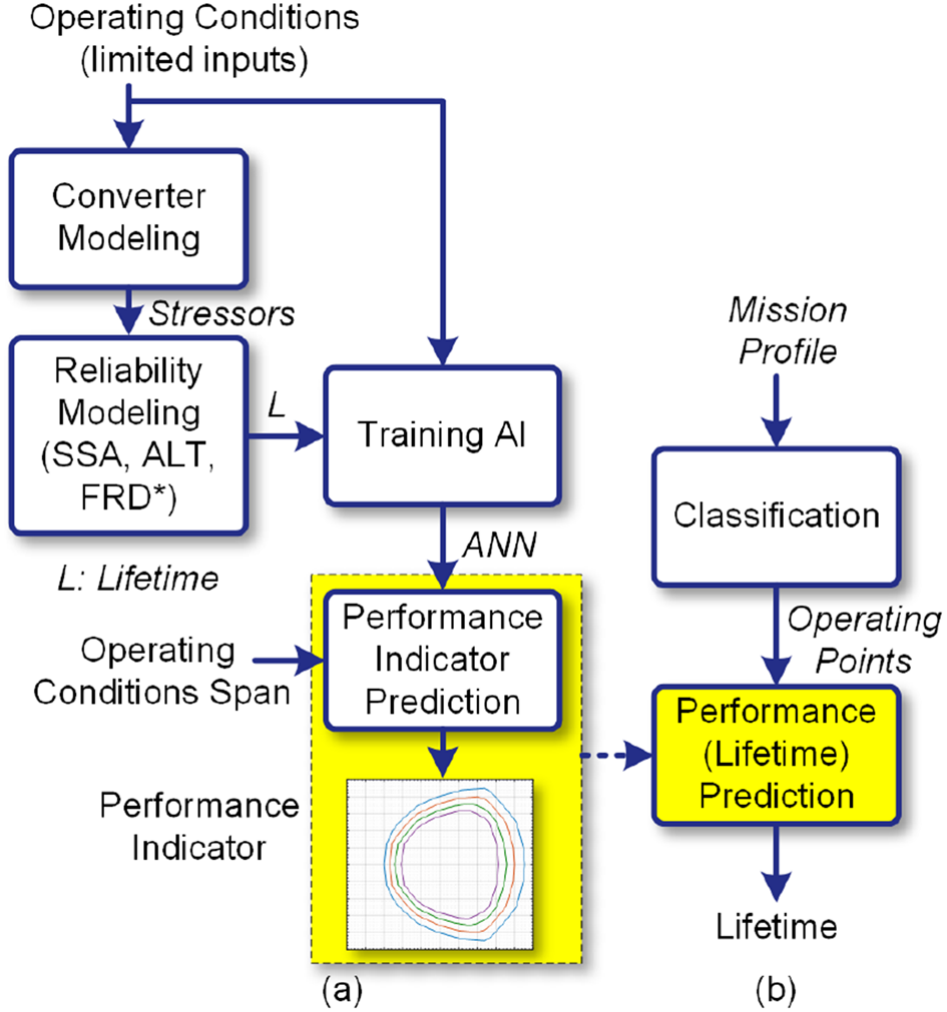
Proposed AI-based long-term performance prediction in converters, (**a**) performance indicator prediction, (**b**) application of performance index in lifetime estimation. *SSA stress-strength analysis, *ALT* accelerated life test, *FRD* field returned data.

Predicting the converter wear-out failure probability for different operating conditions require detailed characteristics and lifetime models of its components. Moreover, lifetime prediction for different operating conditions is time-consuming. In order to facilitate the lifetime prediction in power converters, this paper proposes an AI-based reliability prediction approach for estimating its long-term performance. The proposed approach is shown in Fig. 2. According to Fig. 2a, the converter lifetime, *L* for a limited set of operating conditions is predicted based on the stress-strength analysis. These data can be provided by the manufacturers either using Accelerated Life Test (ALT) or Filed Returned Data (FRD) as well. Then, an ANN is trained based on given operating data and obtained lifetimes. Afterward, the converter long-term performance indicator is predicted for the whole span of operating conditions. This performance indicator can thus be used to predict the converter lifetime operating under different mission profiles as shown in Fig. 2b. In fact, the proposed method is used to predict the converter reliability for a specific application using the limited reliability data provided by the manufacturers (i.e., ALT date or FRD) or SSA with the help of AI. Notably, performing ALT for all operating conditions of a converter is not feasible as a large number of items should be tested for each operating point. Therefore, AI can help to predict the reliability data for the whole operation span. Furthermore, this performance indicator can be implemented in the converter control system for control, operational planning, and maintenance activities as illustrated in Fig. 1.

One of the failure sources causing converter aging is the thermal cycling on power modules^19–21^ as a fragile component of converters^5,21,22,26,27^. As a result, thermal cycling can limit the converter lifetime. The thermal cycles affecting the power module lifetime can be classified into two categories including low-frequency oscillations and fundamental line-frequency oscillations^17,20^. In many applications, the line-frequency impact may be dominating the low-frequency oscillations^20,28^. Line-frequency cycling is a result of the electrical loading of the converter. Therefore, the converter lifetime depends on its active and reactive powers (*P* and *Q*).

**Figure 3:**
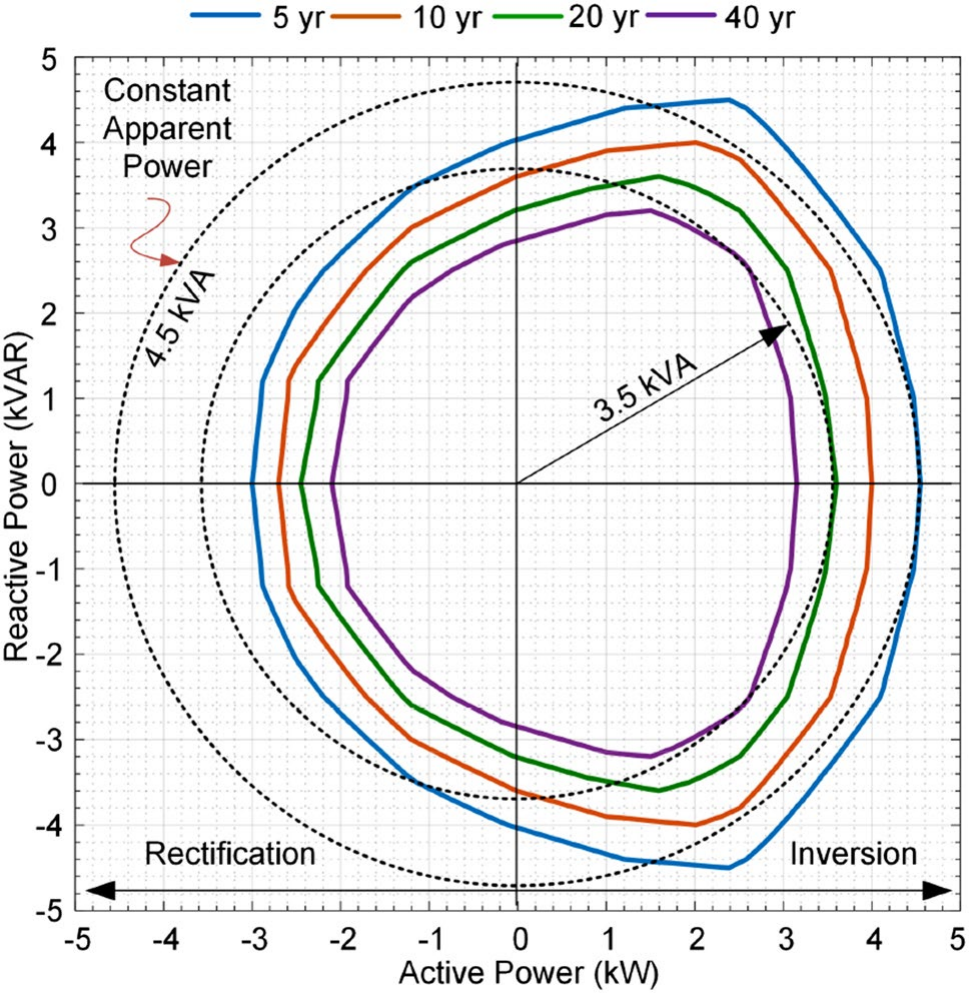
Proposed B_10_ lifetime curves as long-term operation life performance index for a single-phase converter associated with its 90% reliability.

This paper proposes a long-term performance indicator for converters based on its operating conditions (*P* and *Q*) as shown in Fig. 3. This indicator provides constant lifetime curves illustrating the converter lifetime under the different active and reactive power supply. In this paper, the impact of loading is considered. The impact of other parameters affecting the converter lifetime can also be taken into account in the modeling. Similar to other converter characteristics such as efficiency, total harmonic distortion, safe operating area, this index provides another measure of converter performance. Therefore, without analyzing the electro-thermal behavior of the converter, its lifetime can be predicted using these curves. Following this indicator, the converter B_10_ lifetime, the period that the converter reliability is equal to 90%—which can be provided for other B_x_ values—can be predicted following its operating condition. Notably, this indicator is associated with the converter loading, while other operating conditions such as humidity can be included by adding several dimensions to the performance index. This can be used for design for reliability in power electronic-based power systems as well as for system-level economical decision making for planning and maintenance in power systems. In the following, the prediction procedure of lifetime indictor in Fig. 3 is explained. Afterward, the proposed application of AI in converter reliability estimation is presented.

## Reliability prediction in converters

**Figure 4:**
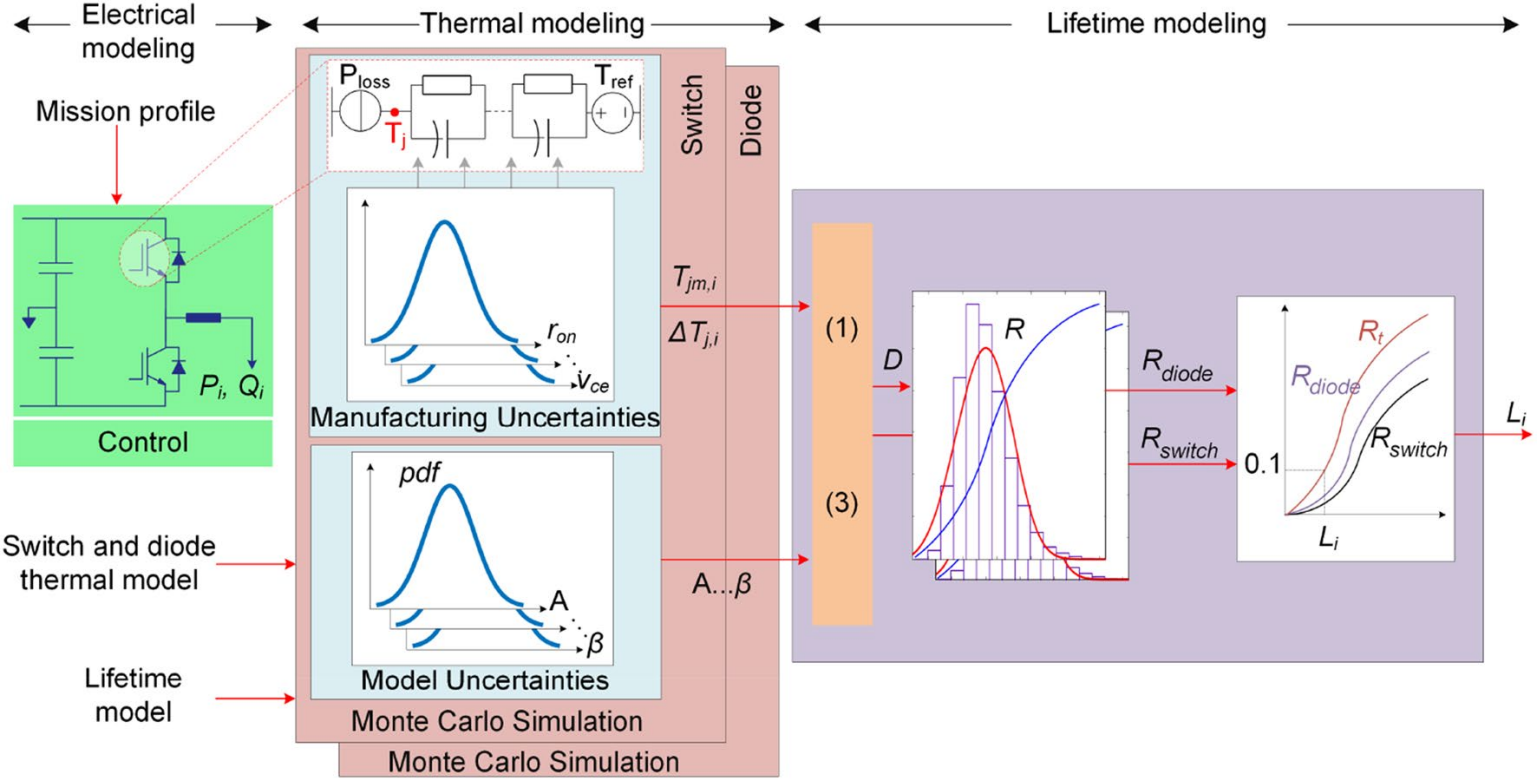
Wear-out failure probability modeling of power modules used in a power converter.

The converter reliability depends on various factors including component characteristics, operation conditions, control and so on. According to the recent field data^6^, power modules are one of the main failure sources in such converters and the power switches are considered to model the overall converter lifetime. The lifetime of power devices can be predicted by the number of thermal cycles to failure, *N*_*f*_ which is given as:

1 $$ N_{f} = A \cdot \Delta T_{j}^{\alpha } \cdot \exp \left( {\frac{\beta }{{T_{jm} }}} \right) \cdot \left( {\frac{{t_{on} }}{1.5}} \right)^{ - 0.3} $$

where, *ΔT*_*j*_ denotes junction temperature swing, *T*_*jm*_ is its minimum value, and *t*_*on*_ is the thermal heating time^29^. According to^29^, *A* = 9.34 × 10E14, *α* = – 4.416 and *β* = 1290. Following (1), the junction temperature and its swing will affect the converter reliability. The junction temperature is associated with the loading of the converter. The converter loading is related to its application and its loading (mission). For instance, the loading of a solar inverter depends on solar irradiance. In order to find the converter lifetime, its electrical loading is transferred to the thermal variables by electro-thermal analysis as shown in Fig. 4. Then, the active and reactive powers are converted to the junction temperature and temperature swing. Afterward, the number of cycles to failure for the given loading condition is obtained by using (1). As already mentioned, the junction temperature swing is divided into low-frequency and line-frequency terms, were the impact of the low-frequency oscillations, in some applications, on power devices lifetime is negligible. Notably, the low frequency oscillations related to the ambient temperature are not considered in this study. Hence, the line-frequency, *f*, oscillations are the main source of the thermal stress. As a result, if a converter operated under *P* and *Q* for a period of *T* (in years), the damage, *D* of the device within *T* can be calculated as $D_{T} = \frac{T \cdot f}{{N_{f,h} \left( {P,Q} \right)}}$. The annual damage, *D* can then be calculated as $D = D_{T} \frac{1}{T}$. The predicted lifetime is equal to the reciprocal of the annual damage. In practice, the device electrical and thermal parameters, such as on state voltage, *v*_*ce*_, on-state resistance, *r*_*on*_, are not identical for different samples of a device, and hence, the predicted junction temperature has to be modified based on manufacturing uncertainties. Moreover, the lifetime model parameters in (1) have some variations. These uncertainties should also be considered in the lifetime prediction. Monte-Carlo simulations can be employed to take into account the uncertainties to predict the device lifetime distribution as shown in Fig. 4^7,18,19^. Therefore, the reliability of power devices, i.e., switches, *R*_*switch*_ and diodes *R*_*diode*_, is obtained as the complementary of their lifetime cumulative distribution function. The total converter reliability is obtained as $R_{T} = \prod\limits_{{N_{s} }} {R_{switch} \prod\limits_{{N_{d} }} {R_{diode} } }$, where, *N*_*s*_ and *N*_*d*_ denote the number of switches and diodes used in converter structure. The converter *B*_*10*_ lifetime under given operating conditions is thus obtained from its reliability function *R*_*T*_.

As shown in Fig. 4, the reliability prediction procedure requires detailed electro-thermal analysis for power devices. Moreover, Monte Carlo simulations are used for considering the impact of manufacturing and model uncertainties. This process not only is time-consuming for different operating conditions, but also requires providing detailed electro-thermal characteristics, device lifetime models and control system parameters such as switching schemes and operating frequency. Moreover, presenting the lifetime curves in Fig. 3 for different operating conditions requires providing huge amount of data from datasheets. Instead of providing detailed electro-thermal, lifetime and control system information as well as the whole lifetime curves in active-reactive power span, the reliability performance index can be provided by the manufacturers for limited operating points. Then, the lifetime at any operating condition can be obtained by employing AI algorithms. This will enable a converter lifetime prediction with limited lifetime data. In the next section, the proposed AI approach for estimating the converter lifetime is presented.

## Proposed AI-based reliability prediction

In order to reduce the computational burden, eliminate the need for detailed electro-thermal and lifetime models, and reduce storing huge amounts of lifetime data, the converter lifetime is predicted using an AI modeling approach. There are various types of AI approaches—and more specifically deep learning approaches—used for modeling non-linear, non-parametric problems including Artificial Neural Network (ANN), Recurrent Neural Network (RNN), Convolution Neural Network (CNN) and so on. The ANN is the basic form of neural networks which maps the input to output in a forward direction. The RNN is a standard form of neural networks, that is designed to recognize sequences. The RNN has been extended across time by having edges feeding into the next time step instead of into the next layer in the same time step^30–32^. Moreover, CNN is a neural network which is designed to recognize images by having convolutions inside for identifying the edges of an object on the image^33–35^. The ANN is much suitable for tabular data analysis, while, the RNN and CNN are suitable for processing and recognition of text, images, audios, videos and so on, where sequence and spatial features are of importance.

In this paper, the AI approaches are used to model the converter reliability based on limited non-linear data. Since the sequence and spatial features are not important, thus, the basic form of neural networks, i.e., ANN is employed in order to predict the converter reliability. The ANN can precisely model non-linear functions between inputs (e.g., active and reactive power) and output (e.g., lifetime). Furthermore, it does not need to store a large amount of data which requires a large memory to store the data. Moreover, it can properly estimate the output associated with the input data out of the data used in the training process. Hence, the ANN becomes a surrogate model for an electro-thermal based lifetime estimator in order to quickly and precisely predict the converter lifetime.

**Figure 5:**
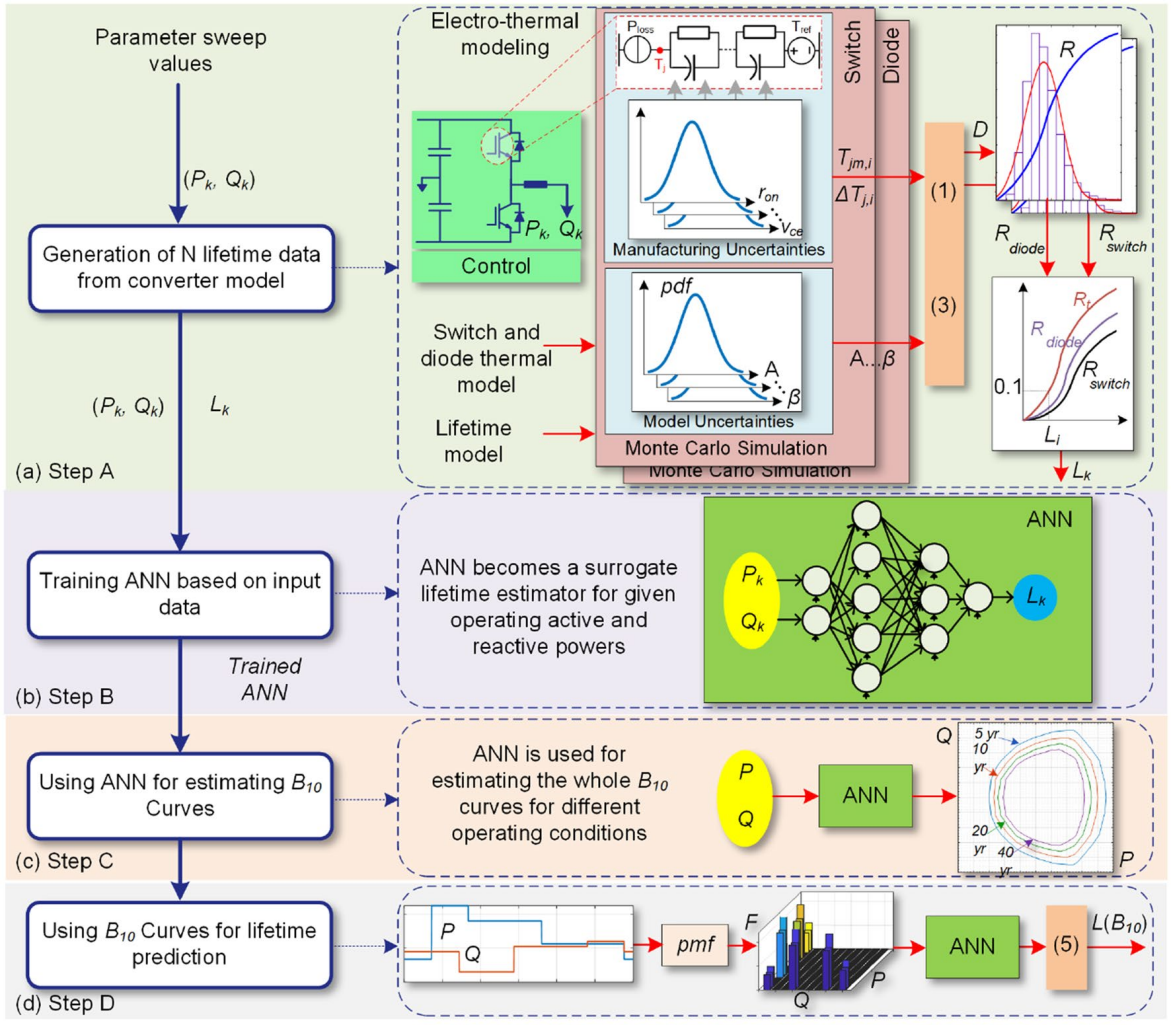
Proposed artificial intelligence-based converter lifetime prediction procedure; (**a**) generating reliability data, (**b**) training ANN, (**c**) *B*_*10*_ lifetime curve estimation, and (**d**) *B*_*10*_ lifetime prediction for a given mission profile. *pmf* probability mass function.

The proposed modeling procedure is shown in Fig. 5, which includes four steps. These four steps include; (*A*) lifetime data generation using electro-thermal based SSA, (*B*) developing ANN, (*C*) predicting converter performance index, and (*D*) predicting converter lifetime, which are explained in the following.

### Step A: Generating limited lifetime data

The first step is to generate a lifetime data associated with the limited operating conditions employing electro-thermal models and SSA as shown in Fig. 5a. This step has been explained in “Reliability prediction in converters”, where a set of limited lifetime data *L*_*i*_ attributed to the active and reactive powers (*P*_*i*_, *Q*_*i*_) are generated. Moreover, these data can also be provided by the manufacturer. In this case, there is no need to provide electro-thermal and lifetime models which might be confidential in most cases.

### Step B: Developing ANN

**Figure 6:**
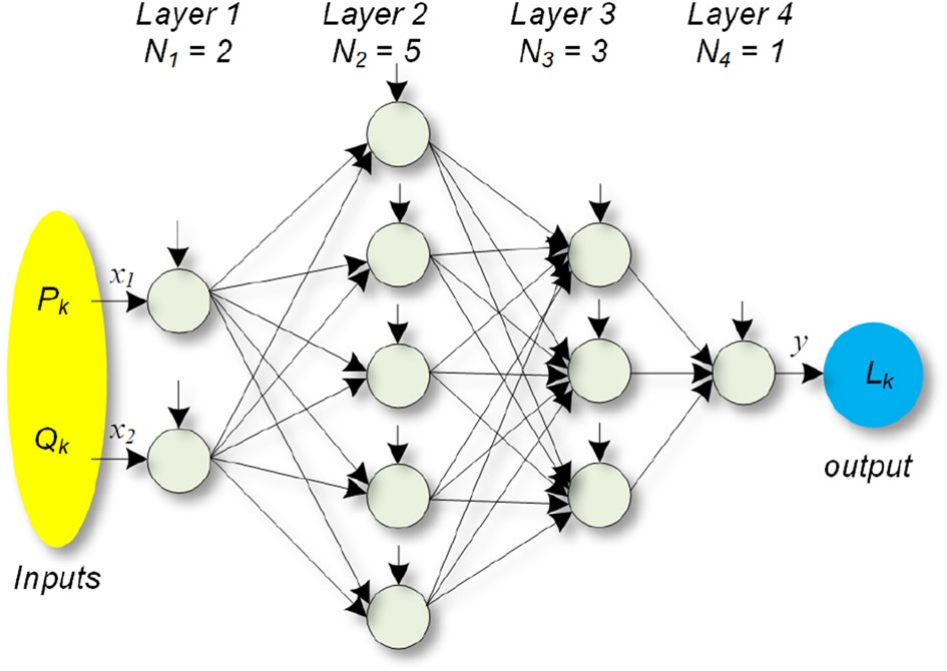
ANN structure. Weights and bias terms are not shown.

The generated lifetime data is used to train the ANN network in the second step as shown in Fig. 5b. Training ANN is carried out by applying the “train” command in the MATLAB Deep Learning Toolbox. The forward ANN comprises several layers, *M* with a number of neurons in each layer *N*_*l*_, *1* ≤ *l* ≤ *M* as shown in Fig. 6. The neurons process the information coming from the neurons of the previous layer to generate the input for the next layer. As a result, the output of each neuron in layer *l*, *1* < *l* < *M* is obtained as^36^:

2 $$ z_{i}^{l} = \sigma \left( {\sum\limits_{j = 1}^{{N_{l - 1} }} {\omega_{ij}^{l} z_{j}^{l - 1} } + b_{i}^{l} } \right)\,\,\,\,\,\,\,\,i = 1,...,N_{l} , $$

where, *σ*(*a*) = 1/(1 + *exp*(*− a*)) is a sigmoid function, *ω*^*l*^_*ij*_ is the weigh between the neuron *j* in layer *l–1* and neuron *i* in layer *l*, and *b*^*l*^_*i*_ is the bias terms of neuron *i* at layer *l*. For layer *l* = *1*,

3 $$ z_{i}^{1} = x_{i} \,\,\,\,\,\,\,i = 1,...,N_{1} , $$

and for layer *l* = *M* (last layer),

4 $$ y_{i} \, = \omega_{i}^{L} z_{i}^{L} = \,\,\,\,\,\,i = 1,...,N_{L} , $$

where, *x*_*i*_ is the *i*th input and *y*_*i*_ is the *i*th output. The inputs are active and reactive powers and thus, *x*_*1*_ = *P*_*k*_, and *x*_*2*_ = *Q*_*k*_ at operating point *k*. The number of layers and neurons can be determined using the try-and-error approach. In this paper, using try-and-error approach, four layers with the number of neurons in each layer equal to *N*_*1*_ = *2*, *N*_*2*_ = *5*, *N*_*3*_ = *3* and *N*_*4*_ = *1* are selected as shown in Fig. 6.

According to^36^, the forward ANN is a universal approximator implying that the weight and bias terms can be selected to obtain the desired resolution in approximating the relation between inputs and outputs. This can be carried out within the training process of the forward ANN employing, e.g., back-propagation algorithm^36^. After training the ANN, it becomes a surrogate lifetime estimator predicting the converter lifetime for a given set of (*P*_*k*_, *Q*_*k*_). Therefore, the converter lifetime can be obtained without using the detailed converter information.

### Step C: Performance index estimation

After generating the lifetime data, and developing the ANN as a surrogate lifetime model of the converter, its performance index can be predicted as shown in Fig. 5c. The converter performance index is its *B*_*10*_ lifetime curves in the operating active and reactive power span as shown in Fig. 3. As already mentioned, obtaining all points in the lifetime curves for different lifetimes using detailed SSA is time-consuming. Therefore, the ANN is used to predict the different lifetime curves associated with different active and reactive power. Hence, the performance index can be obtained by the ANN employing limited lifetime data. This fact will facilitate lifetime modeling by limiting the need for detail electro-thermal analysis and extra information for different application of a converter under different operating conditions. Therefore, the calculation burden will extremely be reduced. Moreover, this performance index will introduce an opportunity for the system-level design of the reliability and optimal decision making in the planning and maintenance of converters. For instance, in the next step (D), application of reliability performance index is presented for lifetime prediction under a given mission profile, which can be used for system-level analysis. A system level application of the proposed AI approach is provided in “Power system-level application”.

### Step D: B_10_ lifetime prediction

The converter lifetime estimation under a given mission profile is performed as the last step like shown in Fig. 5d. For this purpose, active *P* and reactive *Q* power profiles are classified and their frequency is presented by a Probability Mass Function (*pmf*), where *F*_*i*_ is the frequency of (*P*_*i*_, *Q*_*i*_). The converter lifetime associated with each pair of (*P*_*i*_, *Q*_*i*_) is obtained by the performance index curves provided in step C or predicted by using the ANN trained in step (B). Thereby, the *B*_*10*_ lifetime under given mission profile can be predicted as:

5 $$ L\left( {B_{10} } \right) = \left( {\sum {{{F_{i} } \mathord{\left/ {\vphantom {{F_{i} } {L_{i} }}} \right. \kern-\nulldelimiterspace} {L_{i} }}} } \right)^{ - 1} . $$

This equation provides an approximate *B*_*10*_ lifetime of the converter under a given mission profile, which can be used for system-level analysis in power electronics systems.

## Numerical analysis

**Figure 7:**
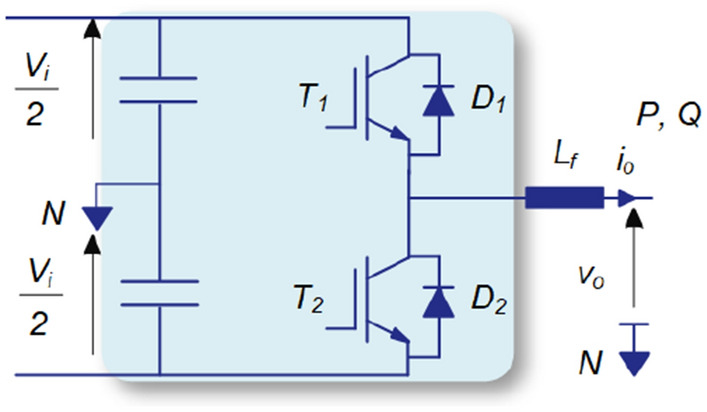
Half-bridge converter used for case studies.

This section presents numerical analysis implying the applicability of AI in power converter reliability prediction. There are various converter topologies used for different applications in power industry. The proposed methodology can be easily applied for any converter topology. Among various converter structures, the half-bridge converter is the building block of many of them. Therefore, without losing generality, a half-bridge converter is considered for analysis as shown in Fig. 7. Later, in the “Power system-level application”, three-phase three-leg converters are considered for reliability analysis in a microgrid. The converter parameters and selected devices are summarized in Table 1. In the following, the impact of converter loading on its performance is demonstrated. Afterward, the application of AI in predicting the converter performance index is presented. Furthermore, the converter reliability operating under different mission profiles are explored employing the converter performance index.

**Table 1:** Converter parameters and devices.

Parameter	Value
Rated power (*kW*)	5
DC voltage (*V*)	400
AC voltage (*V*)	230
Switching freq. (*kHz*)	20
Diode (*D*_*1*_, *D*_*2*_)	IDP30E65D2
Switch (*T*_*1*_,*T*_*2*_)	IGW30N60TP

### Impact of converter loading

**Figure 8:**
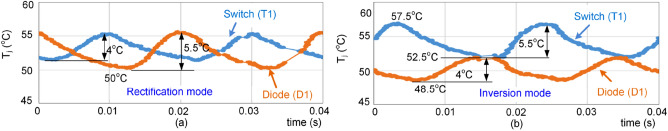
Obtained experimental results showing switch *T*_*1*_ and diode *D*_*1*_ junction temperatures at (**a**) rectification mode, and (**b**) inversion mode.

According to (1), the junction temperature and temperature swing have a significant impact on the converter lifetime. The junction temperature, and consequently, the converter reliability depends on its loading conditions. In order to demonstrate the impact of converter loading on junction temperature of the switch and diode (see Fig. 7), the junction temperatures are experimentally measured under inversion and rectification modes of operation. For experimental measurements, the IGBT module’s cover is and gel is removed and optical fibers are used. The obtained experimental results are shown in Fig. 8a and b for inversion and rectification modes respectively.

As shown in Fig. 8, the minimum junction temperature and temperature swing for both devices, switch and diode, depend on the operational mode of the converter. For instance, the temperature swing of the switch in the rectification mode is 4 °C, while it is 5.5 °C in the inversion mode. Moreover, the minimum junction temperature for diode is 50 °C in the rectification mode and 48.5 °C in the inversion mode. Thus, the operating conditions affect the converter thermal behavior and reliability.

As a result, it should be taken into account during design and operation of converters. Since, the converters are used at different conditions, providing a single number or a curve as a reliability measure in practice is not possible. Therefore, it is crucial to provide the reliability data in such a way that the converter lifetime can be predicted for different operating conditions. This is required for any decision-making within planning and operation of power electronic systems such as in motor drives, renewable power plants, MVDC-HVDC transmission systems, microgrids and so on.

As already mentioned, constant lifetime curves are proposed in this paper to illustrate the converter reliability performance at different operating points as shown in Fig. 3. These data can be provided by converter manufacturers for limited operating points, since covering whole operating conditions is not possible. On the other hand, calculating the reliability data for whole operating region is very time-consuming. Moreover, the reliability prediction using SSA by the power electronic system designers or planners requires detailed lifetime models of its components, which might be not provided by manufacturers due to the market restrictions. Thus, the proposed constant reliability curves for limited points provided by manufacturers is a suitable approach for predicting the converter long-term performance, i.e., reliability under various operating conditions. In the following, the application of AI in using these limited lifetime data in converter performance analysis is presented by several case studies.

### B_10_ lifetime curves as long-term performance index

**Figure 9:**
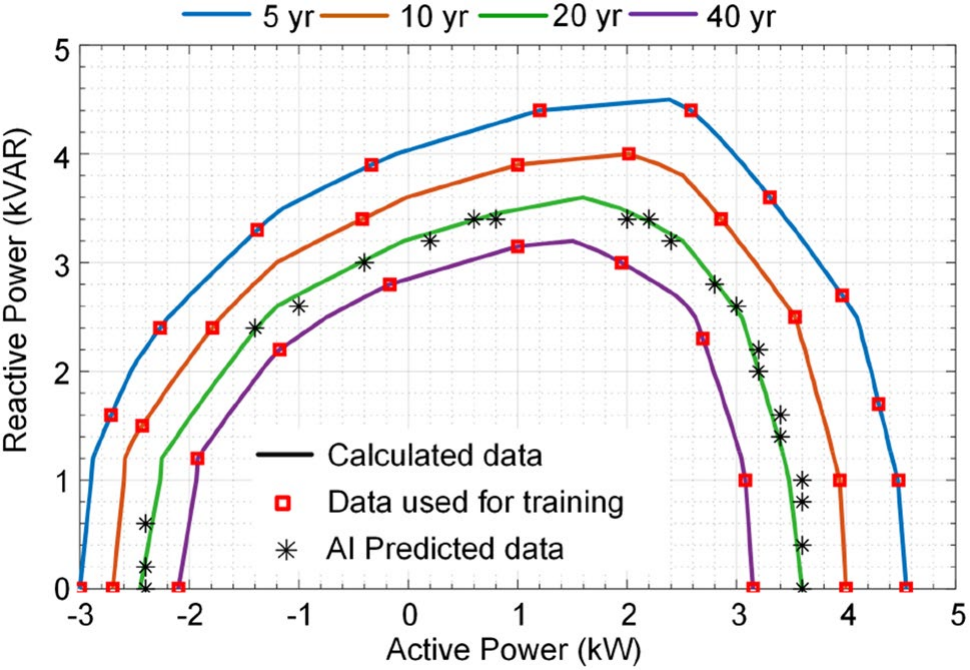
The 20-year *B*_*10*_ lifetime curve prediction based on data of 5-, 10- and 40-year *B*_*10*_ lifetime curves for different operating conditions.

In order to show the performance of the AI application in lifetime prediction, two case studies are provided in the following. The *B*_*10*_ lifetime curves as the performance index of the converter are calculated based on a detailed electro-thermal based SSA as explained in “Proposed long-term lifetime performance indicator”^7^. Then, the 40 operating points (*P*_*i*_, *Q*_*i*_) are selected for training the ANN, which are shown by “**▫**” in Fig. 9. Notably, the solid lines in Fig. 9 are the *B*_*10*_ curves calculated based on detailed electro-thermal model and SSA.

**Figure 10:**
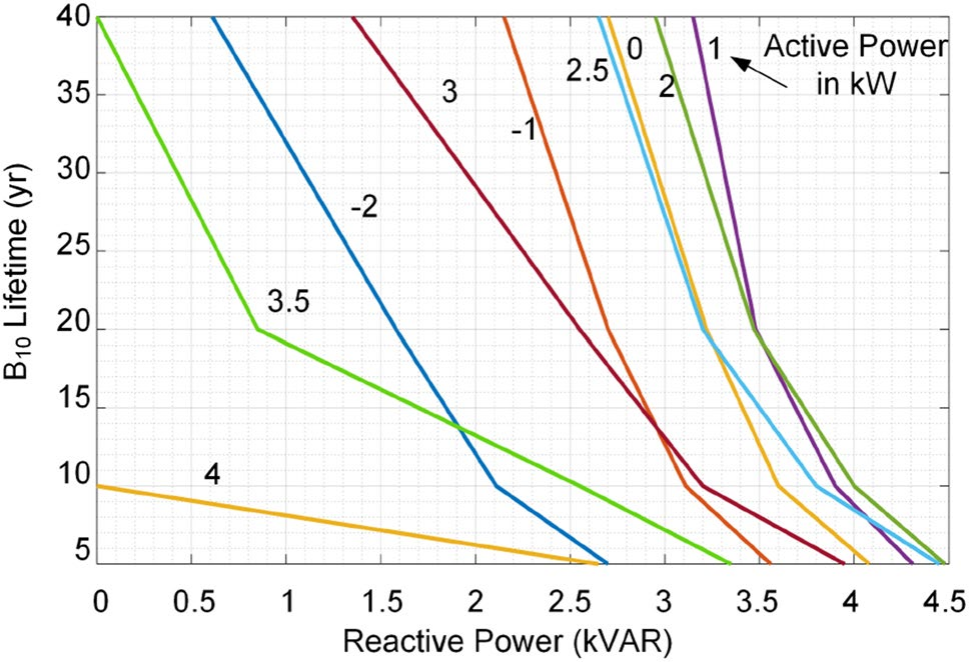
*B*_*10*_ lifetime curves versus reactive power and active power.

The data of 5-, 10-, and 40-year *B*_*10*_ curves highlighted by “**▫**” in Fig. 9 are used to predict the 20-year *B*_*10*_ lifetime curve. The predicted 20-year *B*_*10*_ lifetime data are shown by “*” in Fig. 9, which are very close to the analytical data shown by green line. As it is seen form Fig. 9, the proposed ANN can precisely predict the 20-years *B*_*10*_ curve data using the limited data of the 5-, 10- and 40 years *B*_*10*_ curves. The obtained results show that the AI method can precisely predict the specific *B*_*10*_ lifetime curves by employing limited data of other *B*_*10*_ curves provided by manufacturer. Hence, this will be providing fast lifetime prediction and a simple way to model the converter reliability. Notably, the data correlation given in Fig. 9 is not linear. This means, even the green curve is located in the middle of pink and red curves associated with the 40- and 10-years lifetime, the corresponding lifetime is 20 years instead of being (40 + 10)/2 = 25 years. On the other hand, the distance between curves are almost the same, while the corresponding lifetimes do not change linearly. This non-linear behavior is shown if Fig. 10 in lifetime-reactive power plot for different active powers. It is obvious that there is no clear relation among lifetime, reactive and active powers. Thus, conventional look-up table-based data fitting techniques are not applicable for modeling the converter lifetime curves. However, the AI as a non-parametric surrogate model can properly model the non-linear relation between lifetime and operation conditions. This will be more feasible approach in case more operating conditions such as ambient temperature, low frequency oscillations, etc. which induce more non-linearity.

### B_10_ lifetime estimation under loading profiles

**Figure 11:**
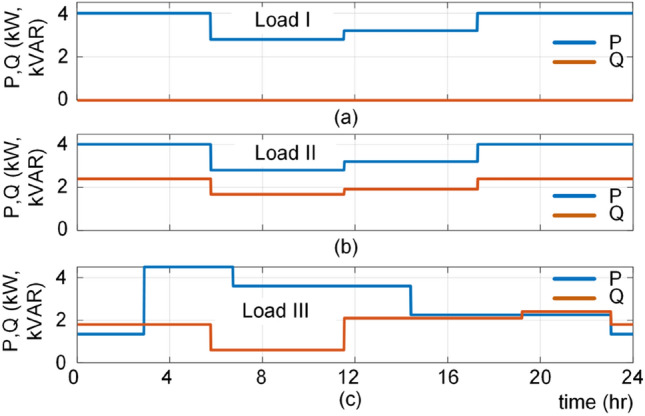
Daily load profiles; (**a**) Load I: Pure active power, (**b**) Load II: Constant power factor, and (**c**) Load III: Non-constant power factor.

In the next case, the lifetime of the converter operating under different mission profiles is predicted employing the conventional approach using the detailed thermo-electrical based SSA model and the proposed ANN approach. Three mission profiles are considered as shown in Fig. 11 including (a) Load I: Pure active load, (b) Load II: Constant power factor load, and (c) Load III: Non-constant power factor load.

**Figure 12:**
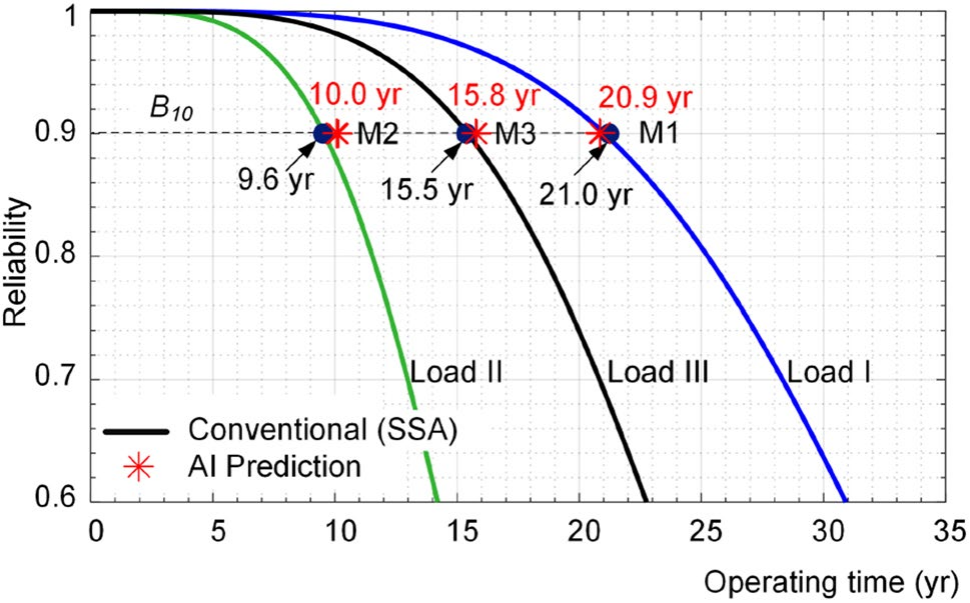
Reliability of converter using conventional SSA approach and AI predicted *B*_*10*_ lifetime for different loading profiles as shown in Fig. 11.

The reliability of the converter is estimated by the conventional approach using analytical SSA model under these three loads and the corresponding reliability functions are shown in Fig. 12. Moreover, the *B*_*10*_ lifetime of the converter for the given load profiles are predicted by the ANN and (5), and the results are depicted in Fig. 12 by “*”. According to Fig. 12, the predicted *B*_*10*_ lifetimes using ANN approach are close to the predicted values by analytical approach using SSA. Thus, it can appropriately predict the converter lifetime under different operating conditions. For instance, as shown in Fig. 12 and summarized in Table 2, the prediction error in points M1, M2 and M3 are 0.5%, 4% and 2% respectively. The obtained results show the effectiveness of the proposed AI-based approach for estimating the lifetime of the converter for different operating conditions.

**Table 2:** Performance comparison between conventional (SSA) and AI-based approach in *B*_*10*_ lifetime prediction for load profiles given in Fig. 11.

Load profile	Predicted points	*B*_*10*_ lifetime (year)			Calculation time (s)	
		SSA	AI	Error	SSA	AI
I	M1	21.0	20.9	0.5	5.43	1.13
II	M2	9.6	10.0	4	5.37	1.12
III	M3	15.5	15.8	2	5.21	1.15

Notably, the conventional approach uses electrical and thermal analyses in time domain, which require detail model parameters of converter components and its cooling system. Moreover, the reliability prediction employs Monte Carlo simulations to predict the failure probability of converters. Not only the electro-thermal analyses and Monte Carlo simulations are time consuming, but also, they need the lifetime model of converter components. In this case, the time consumption for estimating the lifetime under three load profiles are summarized in Table 2 implying five times faster performance of AI based method.

Instead of providing lifetime and electro-thermal models—some data like lifetime models may not be available due to market restrictions—the proposed constant reliability curves can be provided. Furthermore, the proposed AI approach can be used for rapidly predicting the converter lifetime using limited information with an acceptable error like this case study. Notably, the proposed approach is suitable for the applications with slow dynamics of load variations such as electric vehicle chargers and HV/MVDC system converters. Moreover, In the case of fast load variations such as photovoltaic and wind power converters, the proposed approach only provides the lifetime model associated with the line-frequency oscillations^9^. A complete lifetime model requires to include the mission profile by classifying its variations into specific patterns. Thus, the converter lifetime under any mission profile can be predicted by using AI and given lifetime curves for each operational pattern.

## Power system-level application

**Figure 13:**
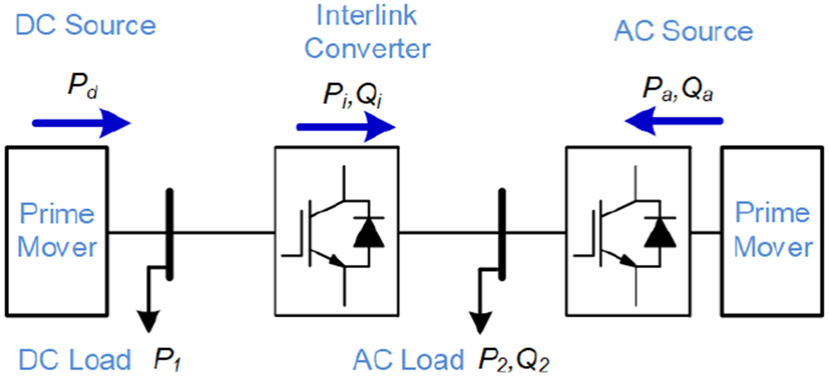
Single line diagram of a hybrid AC–DC microgrid.

This section will present the application of long-term performance indicator in PEPS. In general, the converters are used for interfacing the energy units to the grid, interlinking different sub-girds (either ac/dc, or high/medium/low voltage grids), and driving loads. In this paper, a typical PEPS with a single ac source, single dc source and an Interlinking Converter (IC) is considered as shown in Fig. 13. Moreover, the system loads are localized at common ac and dc busses (dc load: P_1_, ac load: P_2_, Q_2_). This study will focus on the performance of the IC and ac source converter. These converters are identical and have a three-phase three-leg structure, where each leg has the same devices used in Fig. 7 with the rated power of 15 kW.

In this section, the impact of converter application, load sharing and power flow direction will be illustrated on the overall system performance using the proposed AI-based performance indicator. Moreover, the applicability of the proposed indicator in enhancing the overall system reliability is demonstrated.

### Conventional operation approach for hybrid AC–DC grids

**Figure 14:**
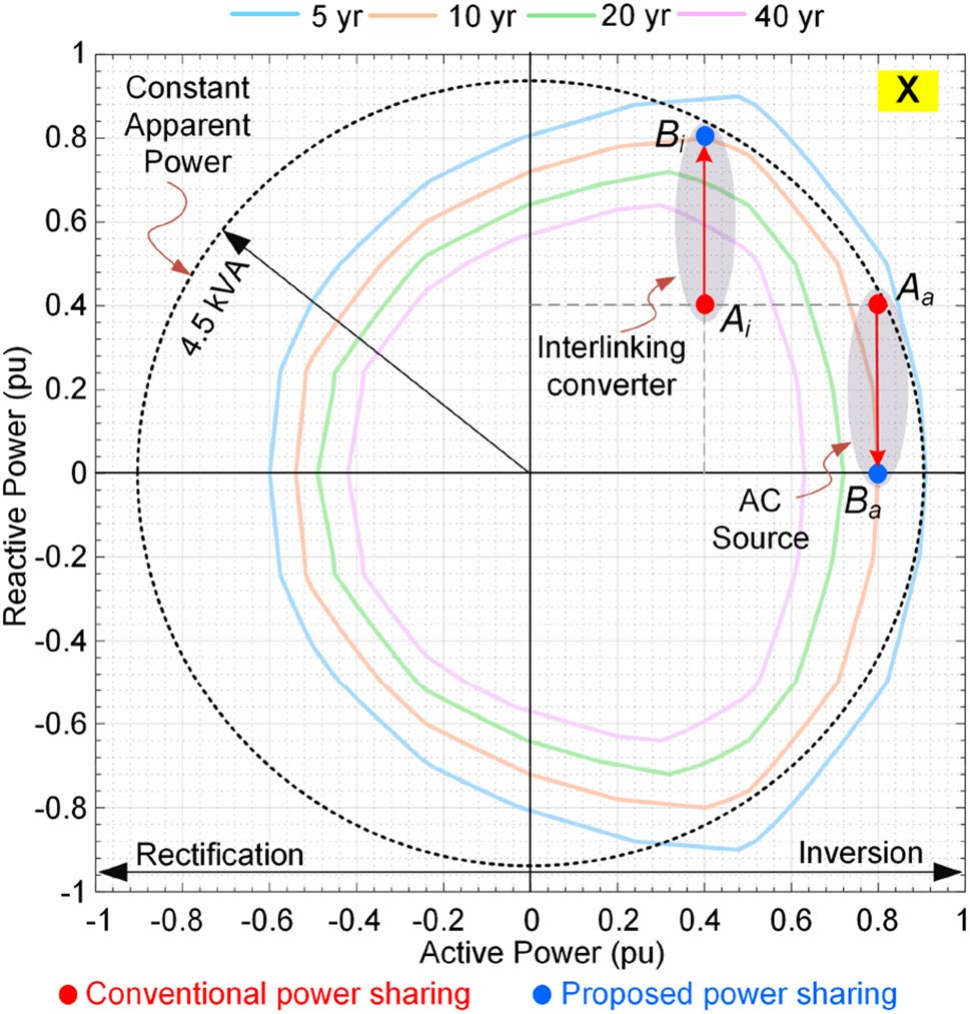
Impact of power sharing on the reliability of converters. P_1_ = 0.4 pu, (P_2_, Q_2_) = (1.2 pu, 0.8 pu).

**Figure 15:**
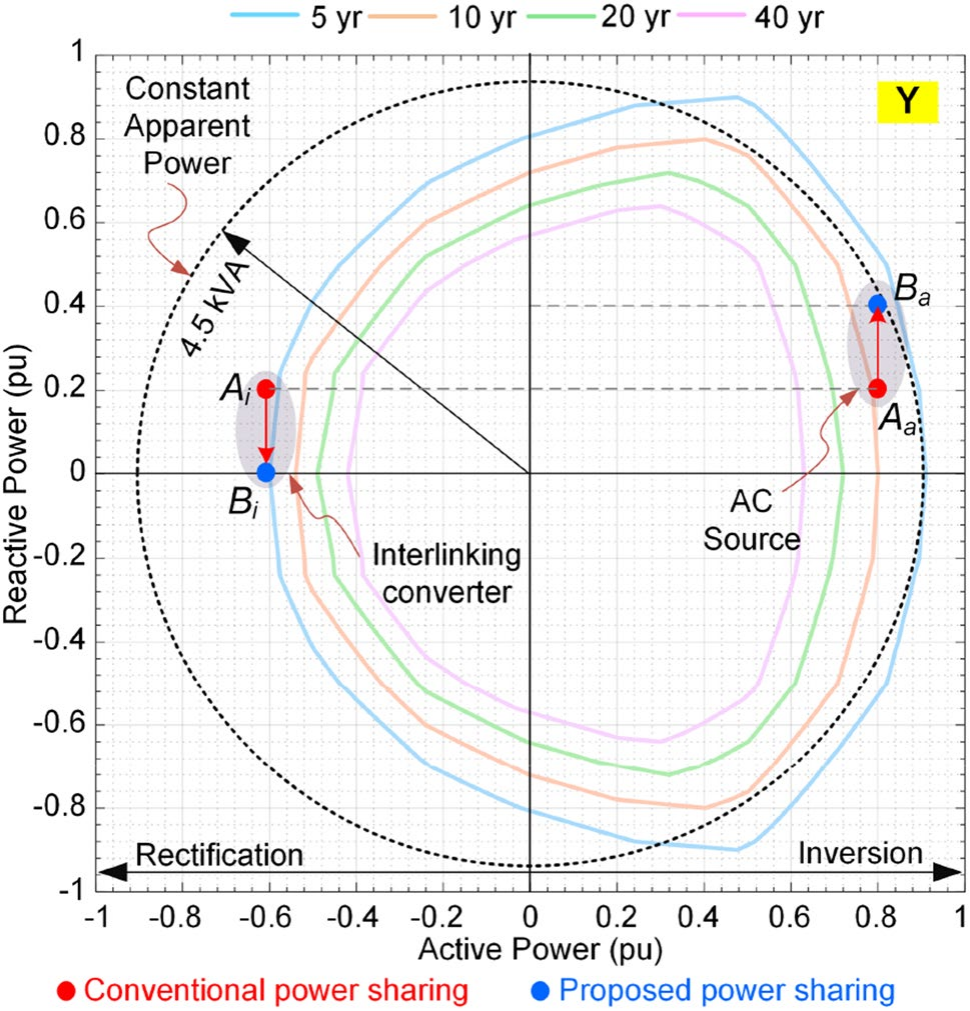
Impact of power sharing on the reliability of converters. P_1_ = 1.4 pu (P_2_, Q_2_) = (0.2 pu, 0.4 pu).

Conventionally, the power sharing between ac sources and dc sources is performed based on their rate powers. Therefore, with an identical rated power for the units shown in Fig. 13, the active power sharing of ac and dc source will be the same. Moreover, the reactive power between ac source and the IC converter is shared according to their rated power. Since, both converters have the same ratings, the reactive power sharing among them will be equal. Technically, the active and reactive power are controlled by droop scheme, which is comprehensively discussed in^37^. In this paper, the focus will be on the reactive power sharing, where according to the droop control method, the output voltage of converters can be controlled with respect to their output reactive power following (6).

6 $$ V_{ref,i} = V^{*} - K_{q,i} Q_{i} $$

where, V^*^ is the nominal voltage, *Q*_*i*_ is the output reactive power, *K*_*q,i*_ is the droop gain, and *V*_*ref,i*_ is the reference voltage. Having the same rated power requires setting the droop gains to an identical value, thus introducing equal reactive power sharing. In the following, two loading conditions are considered including: (a) P_1_ = 0.4 pu and (P_2_, Q_2_) = (1.2 pu, 0.8 pu), and (b) P_1_ = 1.4 pu and (P_2_, Q_2_) = (0.2 pu, 0.4 pu). The base power for per-unitization (pu) is 15 kW. According to the conventional power sharing approach, (P_1_ and P_2_) are evenly supported by the ac and dc sources, thus ac source active power P_a_ = 0.5 × (P_1_ + P_2_). Therefore, the IC’s active power will be P_i_ = P_2_ – P_a_. Moreover, both converters will share the load reactive power equally, i.e., Q_i_ = Q_a_ = 0.5 × Q_2_. These operating points for both loading conditions are depicted on the proposed Q-P plot as shown in Figs. 14 and 15, respectively.

As shown in Fig. 14, the IC has B_10_ lifetime of more than 40 years at point *A*_*i*_ and the ac source converter’s lifetime at point *A*_*a*_ is close to 5 years. Since, the converter operates under rated power, the reactive power sharing can be changed. According to Fig. 14, if the whole reactive power is shifted to IC, the new operating points will be obtained as *B*_*i*_ and *B*_*a*_. It is obvious that the both converters will operate on the B_10_ lifetime curve of 20 years. It is interesting to highlight that the IC is operating close to rated power while the ac converter is below the rated power. However, they both stay on the same B_10_ lifetime curve. This fact shows that the conventional approaches which rely on rated power of converters, cannot guarantee reliable performance of the system. This is because the device lifetime depends on the junction temperature and its fluctuations according to (1). The junction temperature is associated with the average load and the temperature fluctuations depend on the load variations. Considering the rated power for power sharing just impacts the junction temperature. However, in practice, the effect of temperature fluctuations on the device lifetime is dominant^22^.

Moreover, at the second operating condition, the IC is in rectification mode as show in Fig. 15. The operating points of both converters are shown with *A*_*i*_ and *A*_*a*_. The lifetime of IC is lower than 5 years, while the ac source converter has almost 10 years lifetime as shown in Fig. 15. The IC lifetime can be improved by shifting its reactive power to the ac source. In this case, the IC will reach 5-year lifetime curve at point *B*_*i*_ and the ac source will approach 5-year curve at point *B*_*a*_. It is obvious that even though the IC is operating at almost 0.6 pu loading, its lifetime is limited, while the ac source is operating at 0.8 pu loading and it can even support much power to reach the same lifetime curve of 5 years as IC has reached. As the main consequences:

1. The IC has limited lifetime in the rectification mode compared to the inversion mode. Thus, in the rectification mode might be better to work at lower reactive power support to extend its lifetime.
2. These conditions show asymmetric lifetime characteristics of converters; thus, they should be designed considering the lifetime limits either in rectification or inversion mode. That means, a converter with a rated power of 1 pu cannot support 1 pu power in rectification mode (in the present example it can only support 0.67 pu with the same reliability performance operating with rated power in the inversion mode). This is due to the fact that the switches are highly loaded in the inversion mode, while in the rectification mode, the anti-parallel diodes are conducting most portion of the power. Since the electro-thermal characteristics of the diodes and switches are not identical, the lifetime behavior is physically different. If it is operated at the rated power, in both directions, the planning considerations based on rated power of converters are indeed not optimal in practice. This is due to the fact that the converter life expectancy will be lower than the designed value. As a result, the power system maintenance periods may be different from the scheduled times. This is of high importance in modern power systems as the bidirectional converters are growing in the medium voltage dc transmission systems as well as low voltage microgrids. Therefore, precise design of converters considering their lifetime characteristics will introduce optimal planning and operation of future PEPS.

In order to address this asymmetric behavior in the operation of bidirectional converters, the next subsection will propose an intelligent approach to enhance the system performance.

### The proposed approach for lifetime-oriented power sharing

As a result of discussion in the previous subsection for the given two operating conditions in Figs. 14 and 15, it is clear that the routing of reactive power can improve the system reliability. Therefore, the droop equation for reactive power is modified as (7), where *K*_*0*_ is the conventional droop gain and *L*_*i*_ and *L*_*j*_ are the lifetime of *i*^th^ and *j*^th^ converters under the operating points of (*P*_*i*_, *Q*_*i*_) and (*P*_*j*_, *Q*_*j*_). *K*_*q,i*_ is the adjusted droop gain of *i*^th^ converter.

7 $$ K_{q,i} = K_{0} \left( {1 + \frac{{L_{j} - L_{i} }}{{\mathop {Max}\limits_{j \ne i} \left\{ {L_{j} ,L_{i} } \right\}}}} \right),\,\,i,j = \left\{ {1,2} \right\} $$

**Figure 16:**
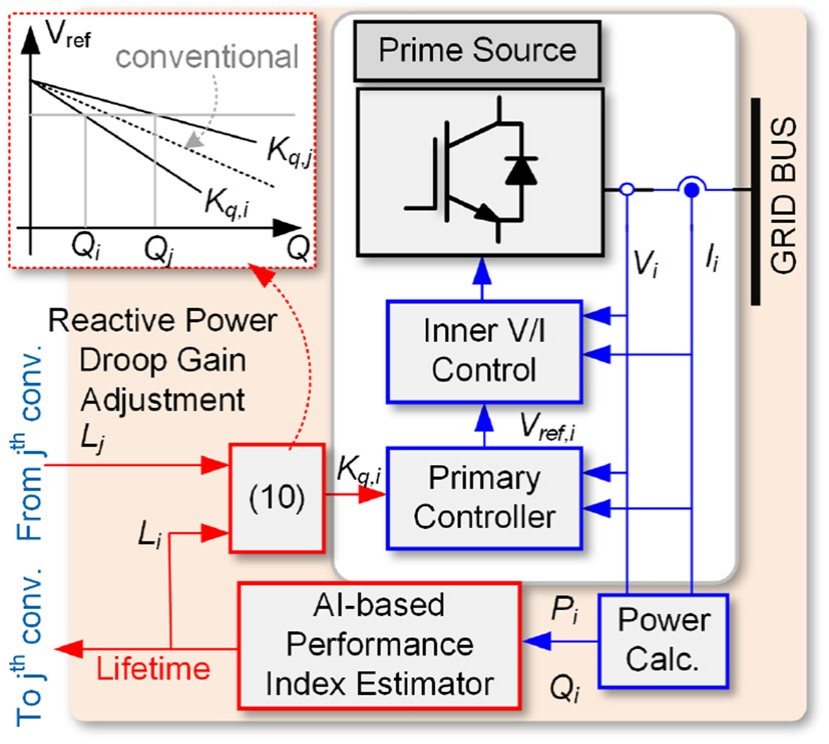
The proposed AI-based power sharing method.

According to Fig. 16, the performance index estimator will predict the lifetime of converter under operating conditions of *P*_*i*_ and *Q*_*i*_ using the proposed AI-based model. The lifetime of converter is compared with the lifetime of *j*th converter. If the *L*_*j*_ > *L*_*i*_, according to Fig. 3, the *j*th converter should take over the majority of reactive power than the *i*th converter. That means, the droop gain of *i*th converter should be larger than that of for *j*th one. This fact is implemented by the adaptive droop gain given in (7), where for the mentioned condition the *K*_*q,j*_ < *K*_*0*_ and *K*_*q,i*_ > *K*_*0*_. Therefore, the droop characteristics for the converters will be different from the conventional one as shown in Fig. 16. As it is expected, using the proposed technique will result in *Q*_*j*_ > *Q*_*i*_. Notably, the reliability-oriented power sharing has already been introduced in^24,38^. However, the employed method was based on the damage of the components instead of using reliability, which requires detail data of thermal and electrical characteristics of devices and heavy computation burden.

**Figure 17:**
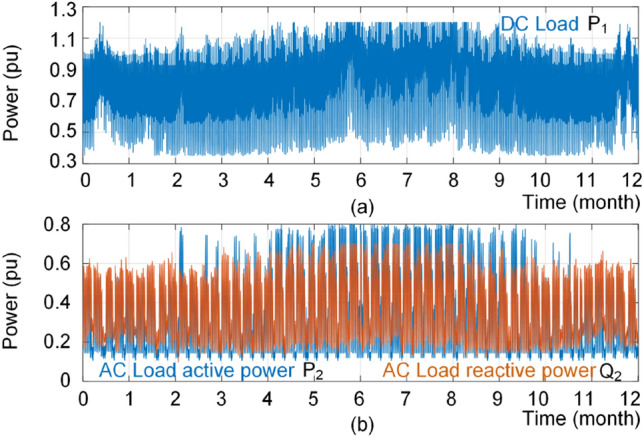
Annual load profiles; (**a**) dc load power, and (**b**) ac load active and reactive power.

**Figure 18:**
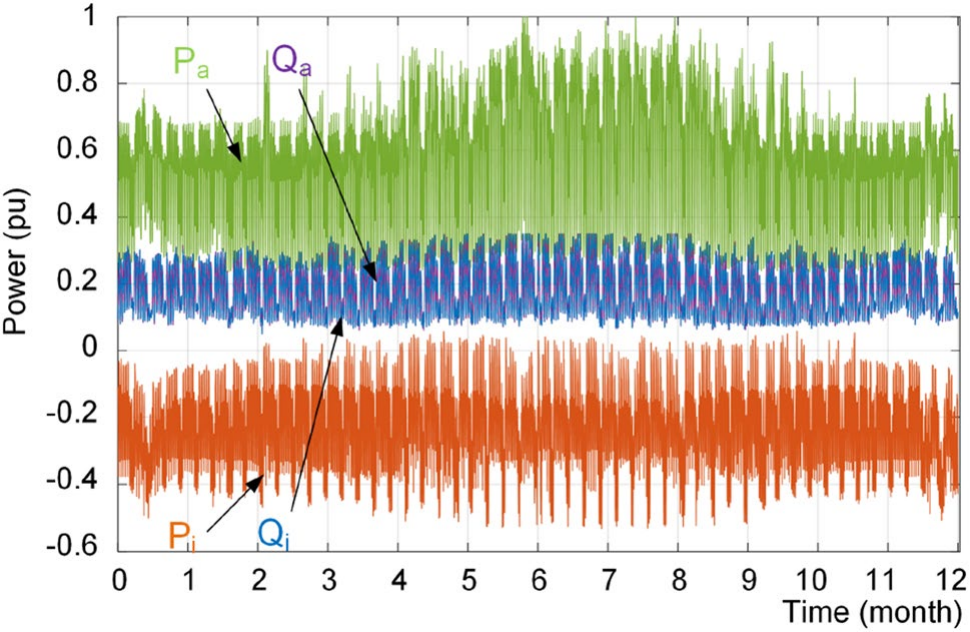
Loading of interlinking converter (P_i_, Q_i_) and ac source converter (P_a_, Q_a_) under conventional power sharing scheme.

**Figure 19:**
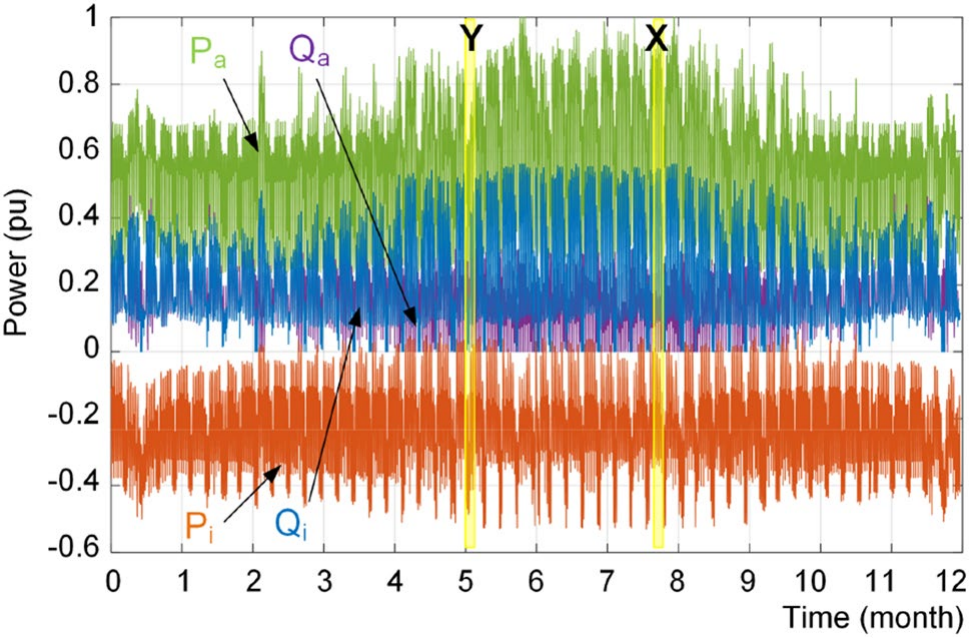
Loading of interlinking converter (P_i_, Q_i_) and ac source converter (P_a_, Q_a_) under reliability-oriented power sharing scheme.

In order to show the performance of the proposed strategy, the dc and ac load profiles are considered as shown in Fig. 17. The result of conventional power sharing is shown in Fig. 18, where the reactive power is shared equally between IC and ac source converter. Furthermore, the IC is mostly operating in the rectification mode. Moreover, the loading of converters using the proposed approach is shown in Fig. 19. It is obvious that the converters have different reactive power sharing. For detail analysis a zoomed graphs of load profile during three days of the year at two point of X and Y are further analyzed. Notably, the X is similar to the loading condition in Fig. 14 and Y is similar to the example shown in Fig. 15 and discussed in previous subsection.

**Figure 20:**
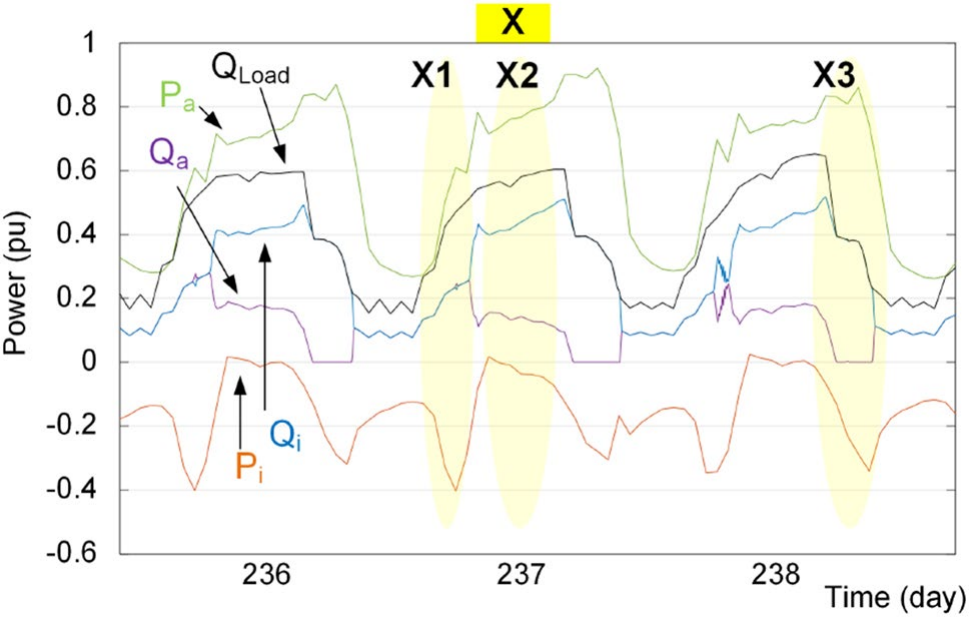
Impact of reliability-oriented power sharing scheme on the converters loading with heaving ac side loading.

**Figure 21:**
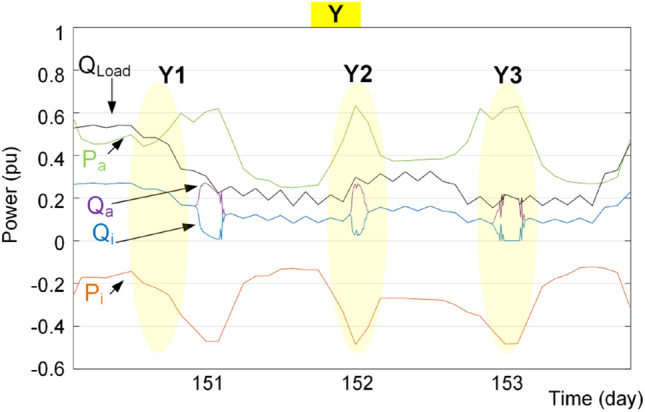
Impact of reliability-oriented power sharing scheme on the converters loading with heaving dc side loading.

According to Figs. 20 and 21, the proposed reactive power sharing process includes three phases. In the first phase (X1/Y1 in Fig. 20/Fig. 21), the reactive powers are equal since the active powers of both IC and ac source converter are much lower than the rated value. The second phase (X2/Y2 in Fig. 20/Fig. 21) shows uneven reactive power sharing as one converter approaching its rated power. In Fig. 20 at X2, the ac source active power is almost 0.8 pu, while the IC is almost transferring zero pu power. Therefore, the IC carries more reactive power than the other converter as shown in Fig. 20. On the other hand, as shown in Fig. 21 at point Y2, the ac source is operating at lower than 0.6 pu, while the IC transfers lower than 0.5 pu. Since at this region according to Fig. 15, the IC approaches its lower lifetime curves, the control system will decrease its reactive power support as shown in Fig. 21. Finally, at point of X3 in Fig. 20, the ac source operates at higher than 0.8 pu that is close to the limited lifetime curves as shown in Fig. 14. Since the IC operates at lower powers, the control system shifts the whole reactive power to the IC to maintain the lifetime of ac source converter. This is similar to the operating condition in Fig. 14 in the rectification mode. Similarly, at point Y3 in Fig. 21, the IC is operating at 0.5 pu, while the ac source operates at 0.6 pu. In this case, the IC is approaching its limited lifetime curves shown in Fig. 15. Thus, the control system shifts all reactive power to the ac source to maintain the IC lifetime.

**Figure 22:**
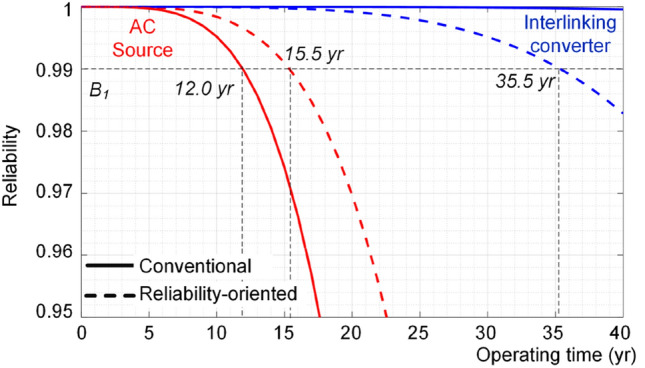
Impact of reliability-oriented power sharing scheme on the reliability of converters.

As a result, the converters lifetime under conventional and proposed power sharing approaches are shown in Fig. 22. The reliability functions are directly predicted using stress-strength analysis shown in Fig. 4 in order to further illustrate the effectiveness of the using the proposed AI-based performance indicator for power sharing technique. To do so, first, the load sharing is performed using the AI-based performance indicator as shown in Fig. 16. Then, the obtained loading profiles are used to predict the converters reliability using the process shown in Fig. 4.

According to Fig. 22, the B_1_ lifetime of ac source is limited to 12 years using conventional power sharing approach, while the IC has a longer lifetime. However, using the proposed approach extends the lifetime of the ac source by 3.5 year (30%), while the lifetime of IC is limited to 35.5 years using the proposed approach. In fact, as shown in Fig. 19, the ac source converter is operating close to limited lifetime curves at much longer times compared to the IC. Therefore, the load reactive power is shifted to the IC as shown in Fig. 19 which decrease its reliability. In practice, if the new lifetime (here 35.5 years) is not acceptable, the system should be re-planned either designing both converters with higher reliability or timely replacing them. It is worth to mention that the results are associated with the wear-out failures. A converter faces different failure types namely random chance, infant mortality and wear-out failures. The two others are extrinsic failures and hence it is not possible to model them theoretically^8^. However, in practice, the lifetime will be lower than e.g., 35.5 years in the last case due to other failure types^7^. Notably, the given analysis and the obtained results are valid for the given load profiles in Fig. 17 and the converter parameters given in Table 1. Different load profiles and sharing coefficient between ac and dc sources will affect the results. This is the main motivation to equipped a converter with a brain to take care of its health. Thus, this paper has introduced an intelligent approach to predict the converter lifetime, which can be used for power system planning purposes.

## Conclusion

This paper has proposed an efficient and compact long-term reliability performance indicator for power electronic converters. This performance indicator is represented by the constant lifetime curves, which are modeled and estimated employing an Artificial Neural Network (ANN). The ANN provides a fast, non-parametric surrogate model for converter performance indicator, which eliminates the need for the detailed electro-thermal and lifetime models of converter components. The proposed performance index can be provided by converter manufacturers as technical data for the optimal and application-specific design for its reliability as well as for operation and maintenance planning in power electronic systems.

The numerical analysis shows that the ANN approach can quickly and accurately predict the converter performance index and its reliability under operating mission profiles. It has been shown that the computation time can be 5 times faster than conventional approach with an acceptable accuracy. Moreover, the application of the proposed ANN approach on the reliable operation of converters with proper active and reactive power sharing in a PEPS has been demonstrated. The proposed power sharing approach introduces 30% lifetime extenuation by considering the lifetime of converter during operation. Moreover, it has been illustrated how the converter behaves in the inversion and rectification mode from reliability standpoint, which should be considered during design and operation of converters. For instance, the lifetime of converter for the selected devices in this paper, was limited in the rectification mode, and operating in that mode in the rated power will extremely reduce its expected lifetime. This paper for the first time introduced a long-term indicator for power electronic converters considering its operating conditions of active and reactive power. Possible future works will be to include the impact of other devices lifetime and fast dynamics of mission profiles. Moreover, other operating conditions such as ambient temperature, humidity and vibration on the lifetime curves of converters could provide comprehensive indicator modeling the converter lifetime.
